# New products

**DOI:** 10.1155/S1463924601000062

**Published:** 2001


Journal of Automated Methods & Management in Chemistry, Vol. 23, No. 2 (March–April 2001) pp. 61–66

### New products

### Genevac launches latest range of evaporation systems at Combinatorial Chemistry 2000

Genevac Ltd is a world leader in solvent evaporation technology for combinatorial chemistry applications. With over a decade of experience in laboratory solvent evaporation applications, it is committed to a large and very active R&D program—the results of which were demonstrated at Combinatorial Chemistry 2000.

Genevac demonstrated its latest range of high throughput evaporation systems at the Show—from the ultra compact HT Series to the large capacity Mega—which feature a number of innovative developments for improved performance and laboratory efficiency, including self-balancing, anti-bumping and continuous running.

*HT-4—Compact for space saving.* Genevac's new Series II HT-4 Systems are designed to provide the ideal solution for solvent evaporation bottlenecks in laboratories using parallel synthesis techniques. The ultra-compact HT-4 centrifugal evaporation system incorporates a space saving built-in condenser and embedded PC for high performance and ease of use.

*Open access evaporator for increased flexibility.* Designed as an integral part of an 'open access' LC/MS purification system for medicinal chemistry, the Genevac HT-4 open access evaporator allows purified fractions to be loaded and unloaded without the delay caused by waiting for a full batch of samples. At any time, additional samples can be loaded into available spaces in the sample rotor and, at every stage in the process, the fractions can be easily identified.

*MEGA—large capacity for high volume.* Genevac's range of Mega centrifugal evaporation systems also offers the efficient use of space—this time combined with extensive capacity.

Designed for large throughput applications. Mega systems incorporate a very large evaporation chamber capable of accommodating large numbers of samples—in modified racks from HPLC fraction collectors (up to 700 tubes per run), proprietary sample formats or microtitre plates (up to 120 deep well plates).

*For more information about Genevac Evaporation Systems and other products please contact: Teresa Picas, Marketing Co-ordinator, Genevac Ltd, Farthing Road, Sproughton, Ipswich IP1 5AP, UK. Tel: + 44(0)1473 240000; Fax: + 44(0)1473 461176; e-mail: teresa picas@genevac.co.uk, URL http://www.genevac.co.uk*

### SAD introduce a new range of compact spectrometers

Spectroscopic and Analytical Developments is a specialized optical design and manufacturing consultancy serving instrument developers, laboratory analysts, process control equipment manufacturers and the pharmaceutical and chemical manufacturing industries.

They develop solutions for on-line and off-line monitoring using spectroscopic techniques in the ultraviolet, visible and infrared wavelength ranges, as well as for other types of optical system.

In order to provide a better service to their customers they have recently developed their own range of low-cost compact spectrometers.

Included in the *MATCHBOX* range are a number of different wavelength ranges and resolutions, thereby making them suitable for a wide range of applications.

Although primarily developed as an OEM product, they are available with an associated electronics module for connection to a PC, and simple software allowing use in research and development applications as well. With a total cost for this system of less than £1000, they are an extremely cost-effective means of spectral analysis.

*For further information plase contact: Dr John P. Ferguson, Spectroscopic and Analytical Developments, 7 Whitechapel Close, Leeds LS8 2PT, UK. Tel: + 44 (0)113 293 1985; Fax: + 1(0)113 228 0204; e-mail: sales@spectroscopic.co.uk*

### Control, flexibility, and compliance in Zymark's MultiDose® G3

Zymark Corporation's newest offering in its distinguished line of fully automated dissolution workstations, the MultiDose® G3 offers control, flexibility and a fully 21 CFR Part 11 compliant platform in one easy-to-use package. The software provides spectrophotometric data acquisition, results calculation, database storage and a comprehensive audit trail. A system administrator can assign various privilege levels to multiple users, permitting different degrees of access to the system's numerous functions. The G3 system can be integrated with a wide variety of third party device drivers, and has the power to create customized reports, making it easier to work with laboratory templates or forms.

The MultiDose family of dissolution worstations brings high levels of automation reliability to dissolution testing needs. All MultiDose products can run multiple methods in a single unattended sequence, making them ideal for the dissolution methods development laboratory, the stability testing group, or for any busy laboratory that needs to run many different products. The systems are designed to require a minimum of operator set-up time so they become productive immediately.

*For additional information contact: Jordana Estner, Marketing Communications. Tel: + 1 (508) 497-6541; e-mail: Jordana. Estner@zymark.com*

### Gene*drive*™: How does it work?

Gene*drive*™ is a revolutionary product released in November 2000 that simplifies PCR testing and greatly speeds it up. Gene*drive*™ and its associated DNA smart*card*™ quite simply offer the fastest PCR analysis in the world.

Gene*drive*™ uses a whole new technology, developed and patented by Dr Ben Cobb, founder and Managing Director of Molecular Sensing PLC. Gene*drive*™ depends on a significant portfolio of patents belonging to the company. The product is small and smart and has been miniaturized to be able to fit into a normal computer—an Apple Macintosh G4. The sample itself is contained, amplified and detected within the confines of a disposable credit card sized device—the DNA smart*card*™.

The fundamental difference between Gene*drive*™ and fluorescence systems is that Gene*drive*™ uses microelectronics to measure the amount of target DNA producted. PCR—Polymerase Chain Reaction—is a standard way of amplifying the amount of DNA prior to identification. Conductivity changes as DNA is produced during a PCR reaction. Gene*drive*™ measures these changes in real time and they are interpreted by special software to give DNA concentration at any given time point. This enables the actual progress of PCR to be mapped over time.

Gene*drive*™ cuts the need for much traditional 'wet' chemistry, which is time consuming and requires skilled technicians to undertake the process. This system is fast and accurate, and also cuts out the expense of using fluorescent monitoring dyes.

Further information about the theory behind Gene*drive*™ can be found on: http://www.molecular-sensing.com

*For further information on* Gene*drive*™*, contact: Dr Michael Bennett, V.P. Sales and Marketing, Molecular Sensing PLC Challeymead Business Park, Bradford Road, Melksham, Wiltshire SN12 8LH, UK. Tel: + 44 (0)1225 706245; Fax: + 44 (0)1225 700056; e-mail: michael.bennett@molecular-sensing.com*

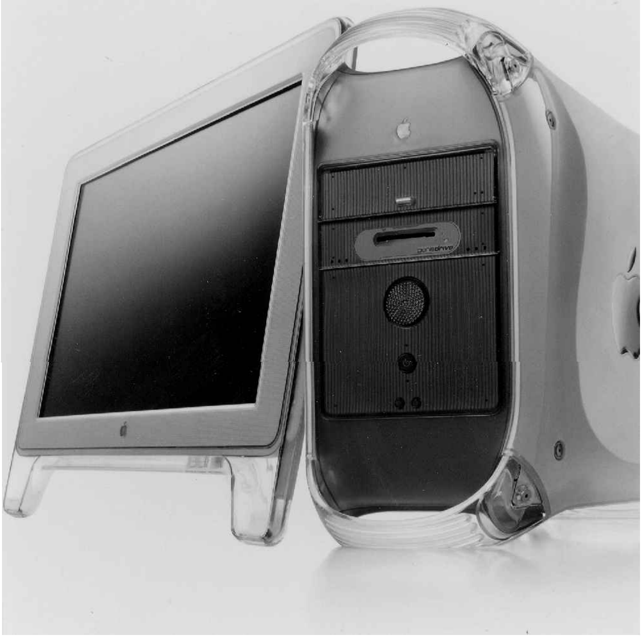

*Molecular Sensing plc PR#398.00* Gene*drive*™.

Gene*drive*™ and DNA smart*card*™ are trademarks of Molecular Sensing Plc. Apple and Macintosh are trademarks of Apple Computer, Inc.

### Draeger adds another four chips to portable gas and vapour monitor for use in the chemicals industry

Draeger has added another four chips to its Chip Measurement System (CMS) range. Ideal for use in the chemicals industry to provide accurate, reliable spot measurements, these new chips enable this portable gas and vapour monitoring unit to measure hydrogen sulphide, ammonia, methylene chloride and styrene.

Requiring minimal user training, the CMS is quick and easy to use. When plugged in to the CMS analyser, which measures and evaluates the concentration, the chips provide an immediate, true digital readout without the need for further evaluation.

Low concentrations of hydrogen sulphide can be found in the production of sodium bisulphide, sodium sulphide, organic sulphur compounds such as thiophenes and mercaptans, sulphate cellulose and sulphur. As a starting compound for many chemical syntheses, such as the production of sulphonamides, chemical fibres, sodium cyanide, nitriles and nitrates, etc., ammonia is also the product of the Haber–Bosch production process using nitrogen and hydrogen. Both of these new chips will measure concentrations up to 0.2–5 ppm.

Methylene chloride is used as a solvent for plastics, propellant for foams, and as an extraction agent. Styrene is produced via direct catalytic dehydration of ethyl benzene, and styrene monomer is a basic component for the production of many thermoplastics. The control of occupational exposure limits, process or product control and the need to check storage vessels, storage tank installations and flanges for their presence, can now be easily accomplished with the methylene chloride chip, which can measure concentrations of 10–200 ppm, and the styrene chip, which can measure up to 2–40 ppm.

Combining intelligent operations with straightforward user instructions, the CMS features a back-lit, multilingual LCD display and a menu-driven user interface. Supplied pre-configured for automatic operation, the analyser performs a self-check on system startup and, as soon as the chip is inserted, carries out a chip integrity test.

Incorporating a mass-flow regulated pump, the CMS measures the movement of concentration in relation to time, regardless of fluctuations in atmospheric pressure. This constant mass flow capability, together with the sensitivity of the reagent system, enables fast, extremely accurate measurements to be made. Once measured, the result is displayed as a concentration on the digital display. In addition, before carrying out a measurement, the system allows the user to select the way in which data is stored.

A tone signal sounds at the end of each measurement cycle and, at this stage, another measurement can be made or another chip selected. A wide variety of chips is available for different applications. Each chip carries a bar code which, when decoded by the analyser, provides the parameters for measurement evaluation, such as the gas type and measurement range. Able to carry out 10 separate measurements, the chips have a two year lifespan.

For those applications where liquids may be present, a special float probe can also be supplied. Protected against dust and spray water in accordance with IP54, the CMS is intrinsically safe and resistant to electromagnetic radiation. It also carries Cenelec (Europe), UL (USA) and CSA (Canada) certification approval.

*Further information is available from Richard Beckwith, Draeger Limited, Ullswater Close, Kitty Brewster Ind Est., Blyth, Northumberland NE24 4RG, UK. Tel: + 44 (0) 1670 352891; Fax: + 44 (0)1670 356266*

### Zymark enhances Allegro™ with new 1536 capabilities

Zymark Corporation is proud to introduce a new High Density Transfer Station Module for the Allegro UHTS System that expands Allegro's functions to include 1536 capability. This versatile and unique addition to the award-winning Allegro is compatible with the complete line of Allegro modules and offers high throughput, flexibility, large storage capacity and increased reliability. The new Transfer Station utilizes Zymark's SciClone™ Liquid Handling Workstation and Presto™ AutoStack plate stacker to provide premier liquid handling and microplate manipulation capabilities that meet the intense demands of both the compound library and UHTS screening.

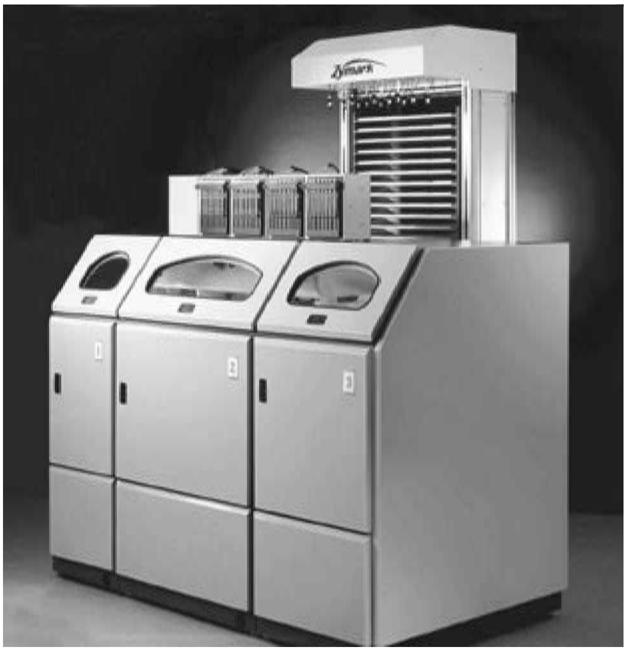

Maximized throughput and flexibility is possible with the High Density Transfer Station's simultaneous loading and unloading of disposables and Allegro's extensive multitasking abilities. The SciClone Workstation adapts to a wide variety of applications, including automatic exchange of pipetting arrays from disposable tips or cannulas, high-speed reformatting from 96 or 384 up to 1536, as well as high-density homogeneous assays. The large storage capacity provided by Presto AutoStack permits unattended operation, and its innovative vertical tray arrangement allows the footprint to remain noticeably compact and manageable.

Zymark's Allegro automation technology is designed specifically to be a high-performance tool for drug discovery, and is constructed with modular workstation units to meet the extreme flexibility requirements of drug discovery research. The system has developed a reputation for operating reliably under the most demanding of circumstances, and is compatible with virtually all conventional assay techniques.

*For additional information please contact: Jordana Estner, Marketing Communications, Tel:+ 1 (508) 497-6541; e-mail: Jordana.Estner@zymark.com*

### Zymark advances liquid handling capabilities

Zymark Corporation has introduced the SciClone™ ALH (Advanced Liquid Handling) Workstation, an exciting and innovative addition to Zymark's prominent line of liquid handlers.

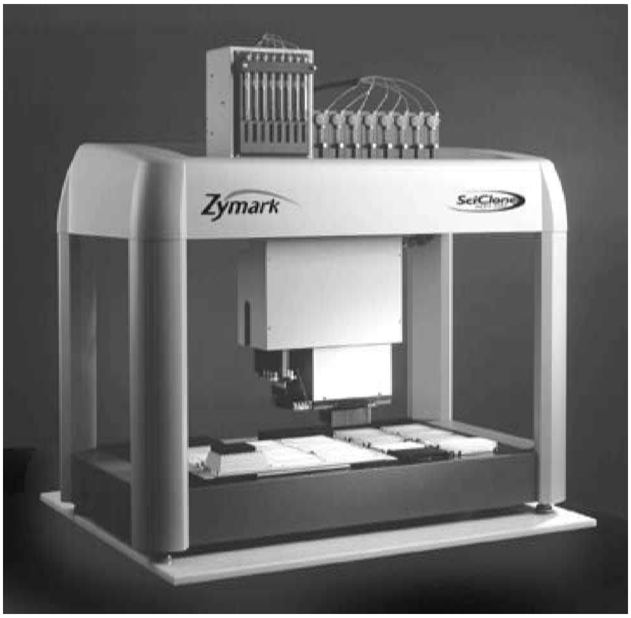

This simple-to-use instrument performs a host of functions that previously required several separate devices, supplying 8-channel independent *Z*-axis probes (Z-8), 'on-the-fly' interchangeable 96 and 384 arrays, a compact 20 position deck, bulk dispensing capabilities and a unique design offering effortless integration with other devices from all four sides of the system.

SciClone ALH's Z-8 probes permit individual access to test-tubes and 96 or 384-well microtiter plates, allowing complete automation of inter-plate and intra-plate formatting for applications such as hit picking, dilutions and tube-to-microtiter plate transfers. The high-density arrays allow inter-plate formatting for up to 1536 well plates, which speeds whole plate replication, formatting and consolidation. The new SciClone ALH simplifies many nucleic acid preparations and plate replications for use in genomic and drug discovery applications.

The hardware and software architecture of the SciClone ALH is specifically designed to ensure evolving laboratory requirements are handled as effortlessly as possible. SciClone ALH utilizes Zymark's much acclaimed CLARA™ software, which has set the industry standard for ease of use and integration.

*For additional information please contact: Jordana Estner, Marketing Communications. Tel: + 1 (508) 497-6541; e-mail: Jordana.Estner@zymark.com; URL http://www.zymark.com*

### Zymark Corporation Launches Staccato™ Workstations

Critical bottlenecks in the drug pipeline identified in the areas of genomics, screening, compound management and ADME/toxicology are placing increased pressure on laboratories. To address these growing challenges, Zymark Corporation announces the launch of its Staccato application workstations.

These application-focused workstations, based on 20 years of automation experience coupled with innovative liquid handling, stacking and control software technologies from recently acquired Scitec, provide a uniform architecture using reliable and economical building blocks. The Staccato workstations expand flexibility, capacity and throughput using 96, 384 and 1536-well microplate technologies.

At the heart of the Staccato Workstations is the Zymark SciClone™ liquid handling and Presto™ autostacking technology, advanced articulated arm robotics and the new CLARA™ 2000 open architecture software. These core automation modules with optional readers, washers, incubators and filtration units gives the user the capability to select the peripherals needed for the application. The result is a scalable, cost-effective workstation with a small footprint and flexibility, throughput and capacity not currently available from any other supplier.

'Two years ago, Zymark launched the Allegro Ultra High Throughput Screening System, which was truly a revolutionary screening breakthrough,' explains Kevin Hrusovsky, Zymark's President and CEO. 'Building on this successful model for standardized automation and additional customer input, we created a broader vision to provide new laboratory automation technology to debottleneck critical applications within the drug pipeline. Since the acquisition of Scitec Automation in 1999, Zymark has successfully incorporated new xyz and articulated arm robotics and scheduling control software that are the cornerstone of the Staccato applications. Eight key application areas in DNA purification, amplification and clean-up as well as ADME toxicology, traditional screening, hit-picking and plate replication all use the same easy-to-use building blocks'.

'Our customers are high profile scientists in key genomic research facilities. They expressed a need for a high-throughput automated workstation to purify plasmid DNA, a very common and time consuming application', commented Roy Vallie, Vice President of Sales and Marketing at Eppendorf 5 Prime. 'Using Zymark's standard liquid handling and storage technologies along with their scheduling software, we have been able to deliver a standard DNA purification platform, compatible with our Perfect Prep-96 chemistry, that reliably delivers 10 times more throughput than any automated platform available on the market—and, more importantly, is out of the box and running in a fraction of the time. Response from customers currently using the workstation has been amazing'.

Zymark Corporation, headquartered in Hopkinton, Massachusetts, USA, is a worldwide leader in laboratory automation for pharmaceutical and biotechnology applications. Zymark serves the worldwide pharmaceutical and biotechnology industries, and hosts the annual ISLAR conference on laboratory automation. The company employs about 300 people in the US, Canada, Japan and Europe, and has authorized sales and service distributors throughout the rest of the world.

*For further information please contact: Jordanna Estner, Marketing Communications, Zymark Corporation, Hopkinton, MA 01748, USA. Tel: + 1 (508) 4976549; e-mail: jordana.estner@zymark.com; URL: http://www.zymark.com*

### Cambridge University confirms the benefits of the Carousel Reaction Station for parallel synthesis

Part of the range of specialist combi-chem systems produced by Radleys Discovery Technologies the Carousel Reaction Station was recently put to the test at Cambridge University.

The Carousel was developed by scientists at GlaxoWellcome to bring the benefits of personal parallel synthesis to organic chemists. Ideal for solution phase and solid supported reagent based synthesis, the Carousel conveniently mounts onto a standard stirring hot plate to convert it into a multiple position reaction station.

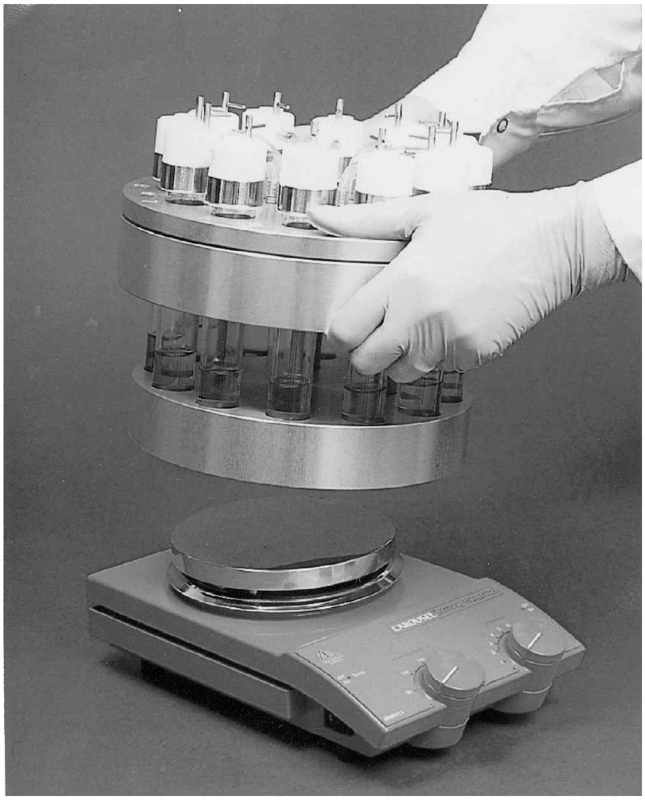

*The Carousel Reaction Station.*

*Cambridge University test report*

In order to assess its performance in practical chemistry applications, Radleys commissioned Dr Baxendale from the Polymer Supported Reagents Group at Cambridge University's Whiffen Laboratory to evaluate the Carousel.

The Carousel tested was the Radleys RR98030 heated/reflux model with IKA stirrer hot plate and digital temperature controller.

A number of practical chemistry requirements were measured:

- the ability to contain solvents at reflux;
- the application to a 'Classical Reaction'—Buchwald coupling chemistry;
- stirring performance tests with solvents of different viscocities, including the addition of solids;
- comparability of different stirring bars.

*Solvent evaporation*

A number of different solvents were tested, heated at a gentle reflux for the prescribed period of time then isolated, removed from the Carousel and allowed to cool before the volume of solvent was measured.

Using a cooling water supply of 7–10 °C, the only solvents that showed significant losses on prolonged heating were the low boiling point solvents—diethyl ether, dichloromethane and to a lesser extent acetone.

When these solvents were retested with the use of chilled water at 0 °C, the solvent loss was reduced to much less significant levels (< 0.5 ml from a 20 ml reaction volume)—even over extended periods of heating (24 hours).

*Classical reaction*

In order to determine the feasibility of transferring chemistry from traditional laboratory glassware to the Carousel, an experiment was carried out based on Buchwald coupling chemistry without any attempt to optimize the yields of the reactions.

2-bromo-p-xylene was chosen as the test substrate and reacted with 1-methylpiperazine and N-methylanaline, which gave 88 and 82%, respectively. These un-optimized yields achieved were comparable with those reported by Buchwald (98 and 94%), clearly demonstrating the ease with which standard chemistry can be transferred to the Carousel.

*Evaluation of PTFE magnetic stirring bars*

Various designs of Radleys stirring bars were tested for their efficiency, including the octagonal, egg-shaped bar and new rare-earth cross-shaped, across a range of viscocities and when heated at reflux.

Whilst both the octagonal and egg shaped bars performed well, even at high revolutions and with the most viscous solution (silicon oil), the new rare-earth cross shaped bar showed significant improvements when compared with other styles of stirring bar.

Dr Baxendale reported: 'This new bar performed well and was found to be far superior in many respects to the other bars tested. Importantly, the more powerful (rare-earth) magnet allowed increased rates of stirring (especially in viscous solutions) and prevented the bar becoming embedded when used in conjunction with solids (including resins)'.

'Our recommendation would be that this new (rare-earth cross shaped) magnetic bar is by far the best design we have tested for use with the Carousel Reaction Station'.

*Practical use in the Whiffen laboratory*

In addition to the official Test Report, Dr Baxendale commented on the use of the Carousel within the Whiffen Laboratory.

'We have utilised the Carousel unit for a variety of chemistry without our group including either polymersupported protocols and more classical solution phase reactions. We have found that the Carousel has enabled us to develop new chemistry much more efficiently. Key advantages to us have been the combinatorial approach the Carousel enables with respect to library generation and reaction optimisation.

'From a practical point of view, the Carousel offers considerable savings on fume cupboard space when compared with using standard laboratory glassware. Additionally, we have found the unit and glassware to be extremely robust, and easy to handle and transport about the lab. The Carousel is also simple to clean and only occasionally requires maintenance of the semi-disposable components.'

*Available worldwide*

The Radleys Carousel is manufactured by Radleys Discovery Technologies and available worldwide through a network of specialist distributors, including RB Radleys in the UK.

*For more information about Radleys Discovery Technologies and its range of specialist Combi-Chem products, please contact: Mark Radley, Sales & Marketing Director, Radleys Discovery Technologies Ltd., Shire Hill, Saffron Walden, Essex CM11 3AZ, UK. Tel: + 44(0) 1799 513320; Fax: + 44 (0) 1799 513283, e-mail: sales@radleys.co.uk; URL http://www.radleys.com*

*For PR information please contact: Jane McCausland or Roberta Bown, Woods Barden Associates, New Road, Stanway, Colchester, Essex, CO3 5HU, Tel: 01206 516006, Fax: 01206 516007, e-mail: woodsbarden@aspects.net*

Editors notes

A fuller article is available on request, including Tables and Buchwald Scheme diagrams. Please contact Jane McCauseland (details above) for a copy.

